# Cytokine and Nitric Oxide-Dependent Gene Regulation in Islet Endocrine and Nonendocrine Cells

**DOI:** 10.1093/function/zqab063

**Published:** 2021-12-01

**Authors:** Jennifer S Stancill, Moujtaba Y Kasmani, Achia Khatun, Weiguo Cui, John A Corbett

**Affiliations:** Department of Biochemistry, Medical College of Wisconsin, Milwaukee, WI, 53226, USA; Department of Microbiology and Immunology, Medical College of Wisconsin, Milwaukee, WI 53226, USA; Blood Research Institute, Versiti, Milwaukee, WI 53226, USA; Department of Microbiology and Immunology, Medical College of Wisconsin, Milwaukee, WI 53226, USA; Blood Research Institute, Versiti, Milwaukee, WI 53226, USA; Department of Microbiology and Immunology, Medical College of Wisconsin, Milwaukee, WI 53226, USA; Blood Research Institute, Versiti, Milwaukee, WI 53226, USA; Department of Biochemistry, Medical College of Wisconsin, Milwaukee, WI, 53226, USA

**Keywords:** beta-cells, cytokines, inflammation, islets, pancreas, single-cell RNA-seq

## Abstract

While exposure to inflammatory cytokines is thought to contribute to pancreatic β-cell damage during diabetes, primarily because cytokine-induced nitric oxide impairs β-cell function and causes cell death with prolonged exposure, we hypothesize that there is a physiological role for cytokine signaling that protects β-cells from a number of environmental stresses. This hypothesis is derived from the knowledge that β-cells are essential for survival even though they have a limited capacity to replicate, yet they are exposed to high cytokine levels during infection as most of the pancreatic blood flow is directed to islets. Here, mouse islets were subjected to single-cell RNA sequencing following 18-h cytokine exposure. Treatment with IL-1β and IFN-γ stimulates expression of inducible nitric oxide synthase (iNOS) mRNA and antiviral and immune-associated genes as well as repression of islet identity factors in a subset of β- and non-β-endocrine cells in a nitric oxide-independent manner. Nitric oxide-dependent expression of genes encoding heat shock proteins was observed in both β- and non-β-endocrine cells. Interestingly, cells with high expression of heat shock proteins failed to increase antiviral and immune-associated gene expression, suggesting that nitric oxide may be an internal “off switch” to prevent the negative effects of prolonged cytokine signaling in islet endocrine cells. We found no evidence for pro-apoptotic gene expression following 18-h cytokine exposure. Our findings suggest that the primary functions of cytokines and nitric oxide are to protect islet endocrine cells from damage, and only when regulation of cytokine signaling is lost does irreversible damage occur.

## Graphical Abstract

**Graphical Abstract:**
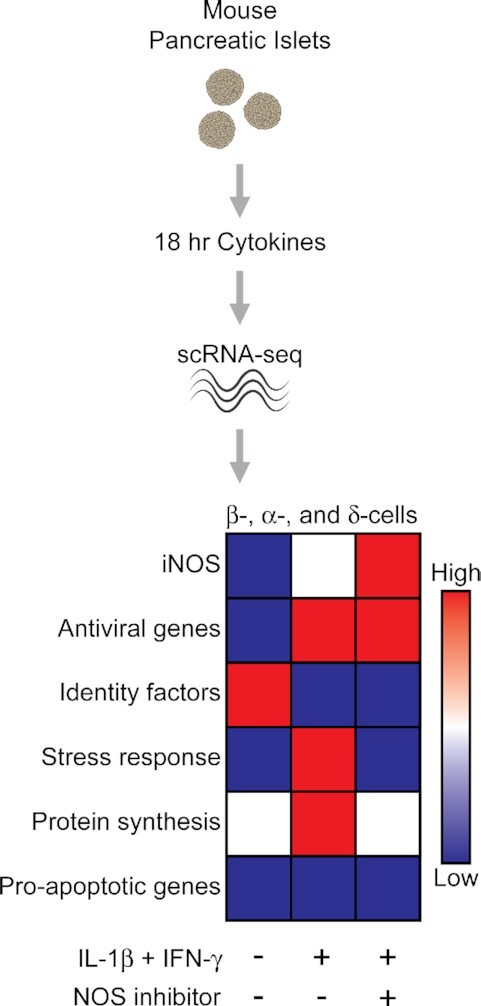

## Introduction

Responsible for synthesis and secretion of insulin in response to a glucose challenge, pancreatic β-cells are essential for survival. Type 1 diabetes (T1D) is an autoimmune disease caused by selective immune-mediated destruction of β-cells, requiring lifelong insulin therapy. While β-cell death in this disease is primarily T-cell dependent ^1–3^, macrophages and T-lymphocytes produce inflammatory cytokines, like interleukin-1 beta (IL-1β) and interferon-gamma (IFN-γ) ^4,5^, which impair β-cell function and induce β-cell death following *ex vivo* treatment and may contribute to disease progression. Specifically, IL-1β inhibits oxidative phosphorylation and insulin secretion and causes ER stress and DNA damage ^6,7^. IL-1β drives nuclear factor kappa-light-chain-enhancer of activated B cells (NF-κB) translocation to the nucleus, which is required for the expression of *Nos2*, the gene encoding inducible nitric oxide synthase (iNOS), and other inflammatory genes ^8–11^. IFN-γ stimulates JAK (Janus kinase)-STAT (signal transducers and activators of transcription) pathways and interferon regulatory factor (IRF)-1 expression and is responsible for the induction of many antiviral genes that limit viral replication ^12–14^. IFN-γ also increases the sensitivity of islets to IL-1β by stabilizing *Nos2* mRNA ^15^. The combination of the two cytokines is required for detectable nitrite formation in mouse and human β-cells ^15,16^. Nitric oxide, generated by iNOS and produced at micromolar levels in β-cells, has been shown to mediate the damaging effects of cytokines ^16–22^.

While IL-1β is thought to solely damage β-cells, recent studies support an alternative primary role of IL-1β and nitric oxide in the protection of β-cells. The expression of a number of antiviral and other protective genes is increased in β-cells following acute (6 h) exposure to the combination of IL-1β and IFN-γ, and a subset of these protective genes is stimulated by IL-1β alone ^23^. Furthermore, nitric oxide protects β-cells from apoptosis and viral infection in a manner that is associated with its ability to inhibit mitochondrial oxidation and deplete cellular energy ^24–26^. Importantly, the inhibitory effects of IL-1β on insulin secretion and DNA damage are reversible ^27,28^, and only after prolonged exposure to the cytokine are the damaging actions permanent ^29,30^. These studies suggest that there are physiological roles for IL-1β signaling in β-cells that are characterized by the expression of protective genes followed by temporary inhibition of mitochondrial oxidation by nitric oxide as a means to protect against environmental threats such as viral or bacterial infection^31^.

Although β-cells make up the majority of the islet (∼70%), other endocrine cells types, like glucagon-secreting α-cells, somatostatin-secreting δ-cells, and pancreatic polypeptide-secreting PP-cells, as well as non-endocrine cell types, like endothelial cells and macrophages, also reside there ^32,33^. The responses of islets to cytokines have been attributed to signaling in β-cells while the responses of other cell types have largely been ignored. This is likely due to the small numbers in which these populations exist and the challenges in isolating these cell types. Additionally, β-cells are thought to be the only islet endocrine cell type that produces nitric oxide following IL-1β exposure, suggesting that non-β endocrine cells may not respond to the cytokine ^22,34,35^. A single-cell approach accommodates the capture and sequencing of multiple cell types within the islet without the need for purification of individual subtypes that is challenging with rare cell populations. This approach allowed us to demonstrate that all islet endocrine cells respond to acute cytokine exposure by increasing *Nos2* and other inflammatory genes, by increasing antiviral and other protective genes, and by decreasing expression of identity genes ^23^. In this first report ^23^, a short cytokine treatment (6 h) was used to examine nitric oxide-independent gene expression. In the current report, we have focused our efforts on nitric oxide-dependent gene expression that occurs in response to a cytokine treatment for a duration at which the actions of cytokines are reversible (18 h).

Here, we utilized single-cell RNA sequencing (scRNA-seq) to determine the cell type-specific effects of nitric oxide signaling in islets and to characterize the heterogeneity of the responses. After exposing isolated mouse islets for 18 h to IL-1β and IFN-γ with or without the nitric oxide synthase (NOS) inhibitor N^G^-Monomethyl-l-arginine (NMMA), we captured and sequenced transcriptomes from over 8000 single cells. Similar to our acute cytokine study, antiviral and other immune-associated genes were increased and identity genes were decreased in all islet endocrine cell types following 18 h cytokine stimulation. The regulation of these genes appeared to be primarily nitric oxide-independent, as addition of NMMA did not attenuate the changes induced by cytokine treatment. We also observed a number of nitric oxide-dependent changes, including increased expression of genes encoding ribosomal proteins, proteins involved in stress responses, and proteins involved in protein biosynthesis. Additionally, we identified iNOS mRNA and protein expression in non-β endocrine cells and nitric oxide-dependent changes in mRNA accumulation that were similar to those observed in β-cells. Finally, cytokine non-responsive cells (those that did not increase *Nos2* or other immune-associated genes) were enriched for heat shock proteins and other chaperones, suggesting induction of a stress response. Our results show that islet responses to cytokines are not unique to β-cells but are similar throughout the entire endocrine population of the islet. Taken together with observations that nitric oxide protects β-cells from apoptosis and viral infection ^24–26^, the studies described here support a model in which a primary function of cytokine signaling in islets is to protect endocrine cells from damage.

## Materials and Methods

### Materials and Animals

Male C57BL6/J mice were obtained from Jackson Laboratories (Bar Harbor, ME) and housed in the Biomedical Resource Center at MCW. All animal use and experimental procedures were approved by the Institutional Animal Care and Use Committees at the Medical College of Wisconsin. Connaught Medical Research Laboratories (CMRL) 1066 medium, Hank's Balanced Salt Solution (HBSS), HEPES, sodium pyruvate, L-glutamine, penicillin, and streptomycin were purchased from ThermoFisher Scientific (Waltham, MA). Fetal bovine serum (FBS) is from HyClone (Logan, UT). Human recombinant IL-1β and mouse IFN-γ were obtained from PeproTech (Rocky Hill, NJ). N^G^-Monomethyl-l-arginine is from Enzo Life Sciences (Farmingdale, NY).

### Islet Isolation, Culture, and Treatment

Pancreatic islets from male C57BL6/J mice, 12–16-weeks-old, were isolated by collagenase digestion and were cultured at 37˚C and 5% CO_2_ in CMRL supplemented with 10% heat-inactivated FBS and containing 5.5 mM glucose as previously described ^36^. Islets from 10 mice per replicate were pooled prior to separation into samples for cytokine treatment. Intact islets were left untreated or were treated with 10 U/mL IL-1β plus 150 U/mL IFN-γ, the combination of the two cytokines plus 2 mM NMMA, or NMMA alone for 18 h before dispersion and preparation for sequencing or before RNA isolation for qPCR. A total of two experimental replicates were used for single-cell RNA-sequencing.

### Single-Cell RNA-Sequencing

Following treatment with cytokines, islets were incubated in 0.48 mM EDTA in phosphate buffered saline and then agitated in 1 mg/mL Trypsin in Ca^2+^/Mg^2+^-free HBSS to disperse into single cells. Cells were filtered and resuspended in HBSS + 0.04% BSA before being loaded into the Chromium Controller (10x Genomics). Single-cell RNA-seq libraries were prepared using the Chromium Single Cell 3′ v3 Reagent Kit (10x Genomics) according to the manufacturer's protocol. Libraries were sequenced using the NextSeq 500/550 High Output Kit v2.5 flow cell (150 cycles, Illumina) with the following conditions: 26 cycles for read 1, 98 cycles for read 2, and 8 cycles for the i7 index read. CellRanger (10x Genomics) functions “mkfastq” and “count” were used to demultiplex the sequencing data and generate gene-barcode matrices (10x Genomics). All scRNA-seq analysis was performed in R (version 3.6.1) using the package Seurat (version 4.0) ^37^. The number of genes detected per cell and the % of mitochondrial genes were plotted, and outlier cells were removed (number of genes less than 200 or greater than 5,500 (replicate 1) or 6,500 (replicate 2), or % mitochondrial genes over 10%) to filter out doublets and cells with low read quality, leaving 10,875 of the original 17,426 cells. Cell cycle genes were regressed. Principal component analysis was performed, and the top 30 principal components were used for Uniform Manifold Approximation and Projection (UMAP) analysis, with clustering performed using the Louvain algorithm. All samples were normalized using Seurat's default normalization settings. Briefly, reads in each cell for each gene were divided by the total number of reads within that cell, multiplied by a factor of 10,000, and transformed using the natural logarithm. Samples from this and our previous study of similar design^23^ were generated in succession rather than in parallel.

### qRT-PCR

An RNeasy kit (Qiagen) was used to isolate total RNA from mouse islets. Thermo Scientific Maxima H Minus reverse transcriptase and oligo(dT)s were used to perform first-strand cDNA synthesis per the manufacturer's instructions. SsoFast EvaGreen supermix (Bio-rad) and a Bio-Rad CFX96 Real-Time system was used to perform quantitative PCR with primers purchased from Integrated DNA Technologies (Coralville, IA). Sequences are listed in Table S1. Gene expression was normalized to *Gapdh* using the comparative ΔCt method for relative quantification ^38^.

### Immunofluorescence Imaging

Mouse islets were treated with 10 U/mL IL-1β and 150 U/mL IFN-γ for 18 h and dissociated as described above. Single cell suspensions were centrifuged onto charged microscope slides using a Shandon Cytospin II (ThermoFisher Scientific). Slides were dried at room temperature for 30–60 min. Cells were fixed in 4% paraformaldehyde for 15 min, permeabilized with 0.1% Triton X-100 in PBS for 30 min and blocked using 1% BSA in PBS-T (0.2% Tween-20) for 1 h at room temperature. Primary antibodies were diluted in 1% BSA in PBS-T and were incubated overnight at 4°C. Secondary antibodies were diluted in 1% BSA in PBS-T and were incubated for 1 h at room temperature. Antibodies and dilutions were as follows: rabbit antiiNOS (Cayman Chemical, 1:1000), sheep antisomatostatin (American Research Products, 1:1000), mouse antiglucagon conjugated to Alexa Fluor 488 (Santa Cruz Biotechnology, 1:100), donkey antisheep conjugated to Alexa Fluor 488 (Invitrogen, 1:1000), and donkey antirabbit conjugated to Cy3 (Jackson Immunoresearch, 1:1000). ProLong Gold Antifade Mountant with DAPI (ThermoFisher) was added to slides prior to addition of coverslip and imaging. Grayscale images were captured using a Nikon eclipse 90i confocal microscope and were pseudo-colored using ImageJ (National Institutes of Health).

### Statistical Analysis

For differential expression analysis, *P*-values were calculated using the Wilcoxon test, and Bonferroni correction was used to avoid false positives. An adjusted *P*-value < 0.05 was the threshold used to declare significance. For qPCR analysis, GraphPad Prism software was used to make statistical comparisons between groups using one-way ANOVA with Šídák multiple comparison test, and *P* < 0.05 was the threshold used to declare significance.

## Results

### Single-Cell RNA-Sequencing of Mouse Islets Following 18 h Cytokine Treatment

**Figure 1.**
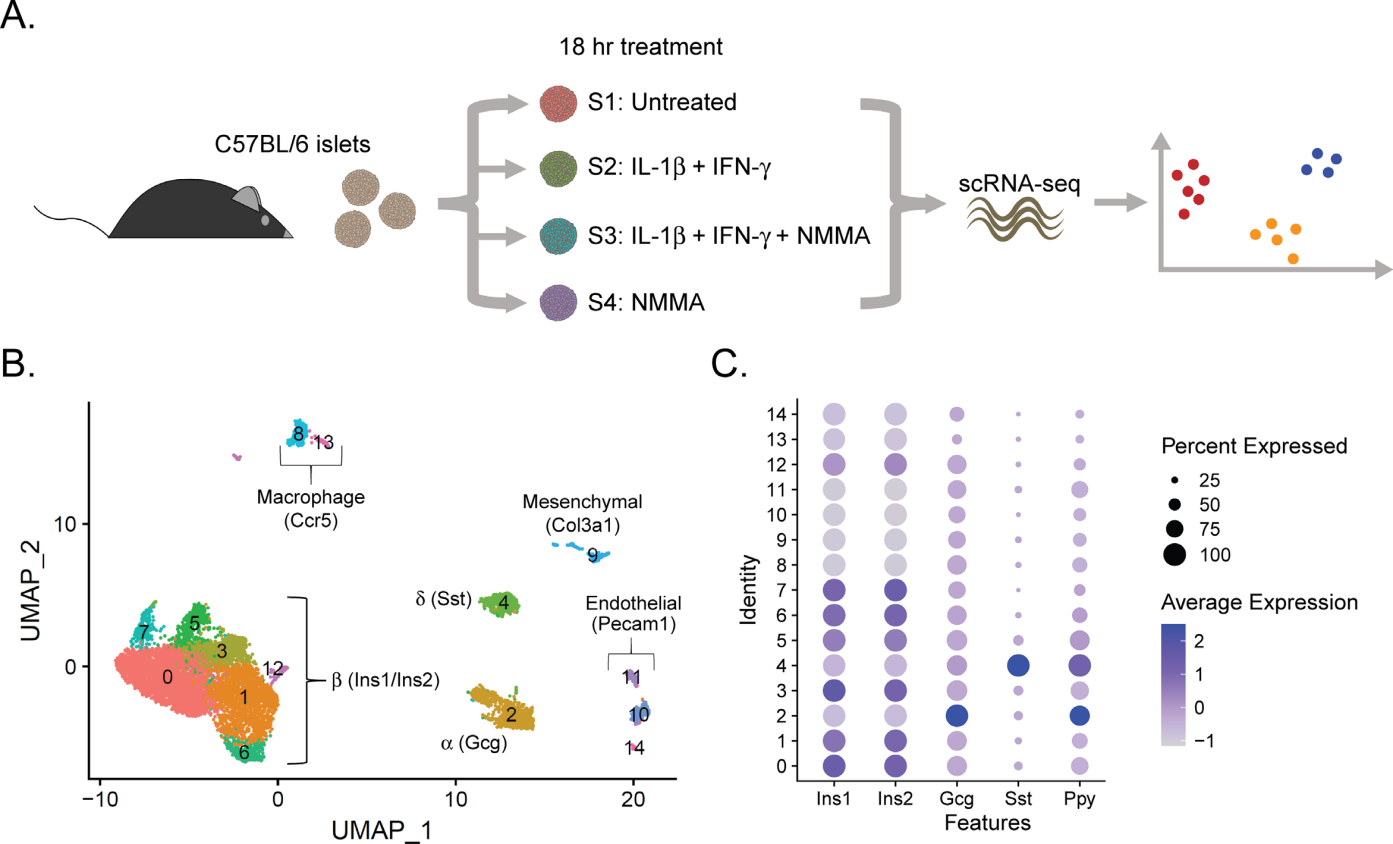
scRNA-sequencing of mouse islet cells after 18 h cytokine exposure. (A) Schematic of experimental design. (B) UMAP plot depicting clusters of cells from both scRNA-seq experimental replicates. Cell identity was assigned based on enrichment for genes indicated. β-cells (*Ins1* and *Ins2*) comprised 74% (6269 cells) of our dataset, α-cells (*Gcg*) 10% (854 cells), and δ-cells (*Sst*) 6% (488 cells). (C) Dot plot indicating expression levels and % of cells expressing *Ins1, Ins2, Gcg, Sst*, and *Ppy* mRNA in each of the 15 clusters. Concentrations are as follows: 10 U/mL IL-1β, 150 U/mL IFN-γ, and 2 mM NMMA.

Expression of iNOS in mouse β-cells requires stimulation by both IL-1β and IFN-γ ^15,16,39^. To determine how cytokine-derived nitric oxide influences gene expression changes in the different populations of islet cells, we performed scRNA-seq using mouse islets. Samples were untreated (Sample 1) or treated for 18 h with 10 U/mL IL-1β + 150 U/mL IFN-γ alone (Sample 2) or with 2 mM NMMA (nitric oxide synthase inhibitor; Sample 3), or with NMMA alone (Sample 4; Figure 1A). Cells from all samples and both experimental replicates were visualized using UMAP, an algorithm that unbiasedly grouped the cells into 15 clusters based on similarity of gene expression (Figure 1B and Table S2). Cells from both experimental replicates populated the clusters approximately equally (Figures S1A and S1B). We assigned endocrine cell identities (β-, α-, and δ-cells) based on enrichment of the primary islet hormones (insulin, glucagon, and somatostatin, respectively; Figure 1C). β-cells (*Ins1* and *Ins2*) comprised 74% (6269 cells) of our dataset, α-cells (*Gcg*) 10% (854 cells), and δ-cells (*Sst*) 6% (488 cells). We were unable to separate pancreatic polypeptide-expressing cells from the *Gcg*- and *Sst*-expressing clusters (Figure 1C). Using characteristic gene expression, we also identified the cell types of the non-endocrine clusters, which each made up less than 4% of our dataset: endothelial cells (*Pecam1*), macrophages (*Ccr5*), and mesenchymal cells (*Col1a1*; Figure 1B).

### Types of Genes Changed in β-cells in Response to Cytokines

**Figure 2.**
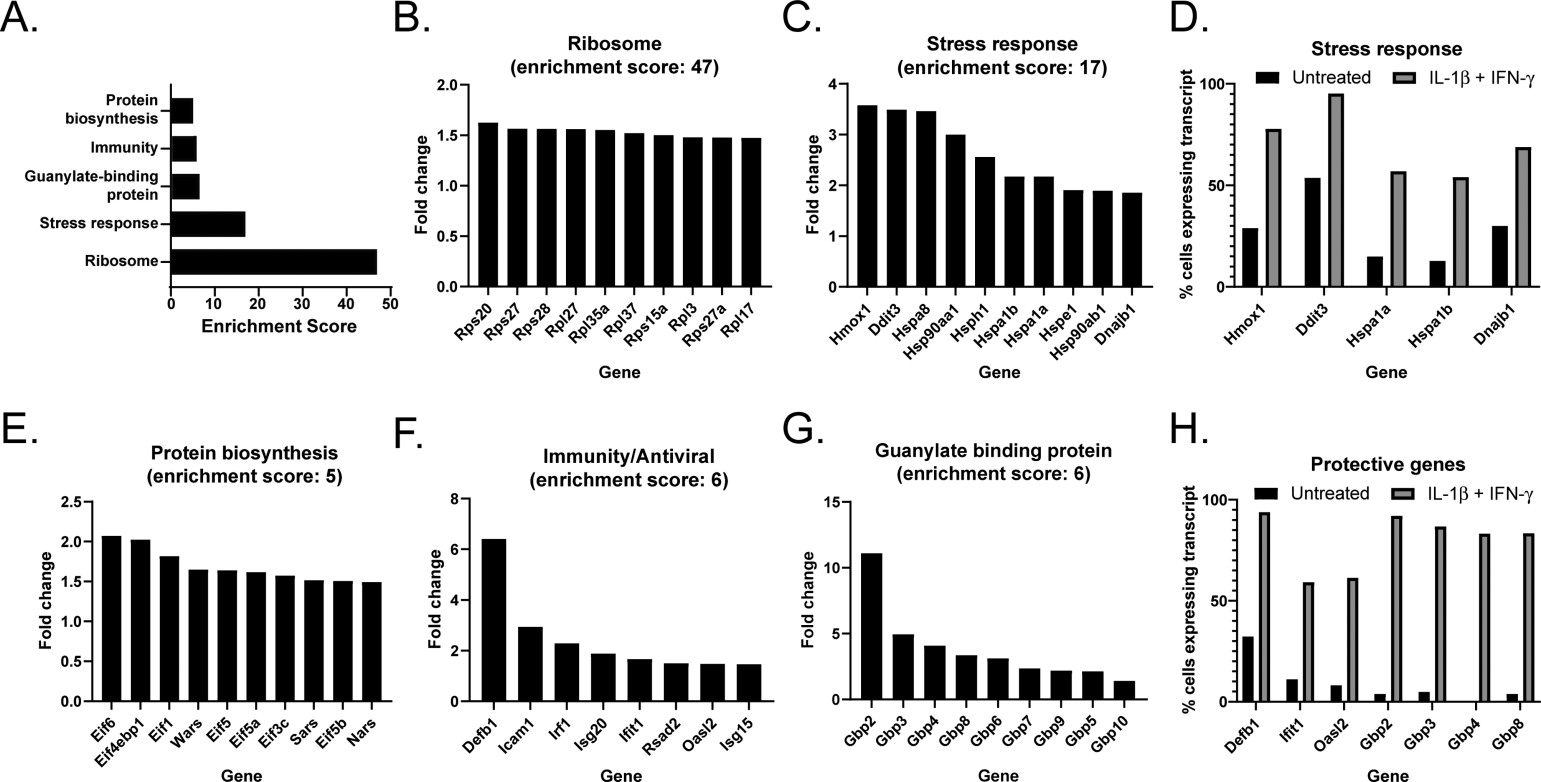
Categories of genes changed in β-cells following 18 h cytokine treatment. (A-C and E–G) Genes that were significantly different in β-cells following 18 h cytokine stimulation compared to untreated β-cells by scRNA-seq were subjected to functional annotation clustering using the Database for Annotation, Visualization and Integrated Discovery (DAVID). Selected enriched categories of genes are shown in (A). Fold change (over untreated) of representative genes in the selected enriched categories of Ribosome (B), Stress response (C), Protein biosynthesis (E) Immunity/antiviral (F), and Guanylate binding protein (G) are shown. (D and H) Percent of β-cells expressing selected genes involved in stress response (D) or protection (H) in the untreated or cytokine-treated conditions.

To better understand how β-cells respond to cytokine exposure, we computationally isolated Clusters 0, 1, 3, 5, 6, 7, and 12 from the total dataset (Figure 1B) and performed differential expression analysis to determine genes significantly changed in response to each treatment (compared to the untreated sample). In total, 1272 genes (773 increased and 499 decreased) were significantly changed in β-cells in response to 18 h cytokine stimulation (Table S3). To get an unbiased view of the categories of genes in this dataset, we used the Database for Annotation, Visualization, and Integrated Discovery (DAVID) to identify enriched categories of genes increased in β-cells in response to cytokines (Figure 2A) ^40^. By far, the most enriched category contains genes encoding ribosomal proteins (Figure 2B). Genes in this category comprise ∼10% of the genes increased by cytokines in β-cells (77 of the 773). Genes involved in cellular stress responses, including heat shock proteins, heme oxygenase 1 (*Hmox1*), and DNA damage inducible transcript 3 (*Ddit3* or *Chop*), which is associated with the unfolded protein response, are also increased, as expected (Figure 2C) ^41–43^. Not only is the expression level of stress response genes increased following cytokine stimulation, but the percentage of β-cells expressing those genes is also increased (Figure 2D). Genes involved in protein biosynthesis, including several eukaryotic initiation factors and tRNA synthetases, are also increased (Figure 2E). We have previously observed increased expression of genes encoding proteins with antiviral/antimicrobial properties in islet endocrine cells (β-, α-, δ-, and PP-cells) following acute exposure (6 h) to inflammatory cytokines ^23^. In agreement, genes in this category remain elevated following 18-h cytokine treatment, including *Defb1, Rsad2*, and *Oasl2*, and multiple guanylate binding proteins (Figure 2F and G). The percentage of β-cells expressing these protective genes increased as well, with several antiviral genes being expressed in greater than 80% of β-cells in response to cytokines while being expressed in less than 5% under basal conditions (Figure 2H). Together with the observation that pro-apoptotic genes are not increased in β-cells after 6 h ^23^ or 18 h cytokine treatment (Figure S2), in contrast to the suggestions of others ^44^, the increase in protective gene expression suggests that the primary response of β-cells to cytokines is protective rather than damaging.

### Nitric Oxide-Dependent Gene Expression Changes in β-cells

**Figure 3.**
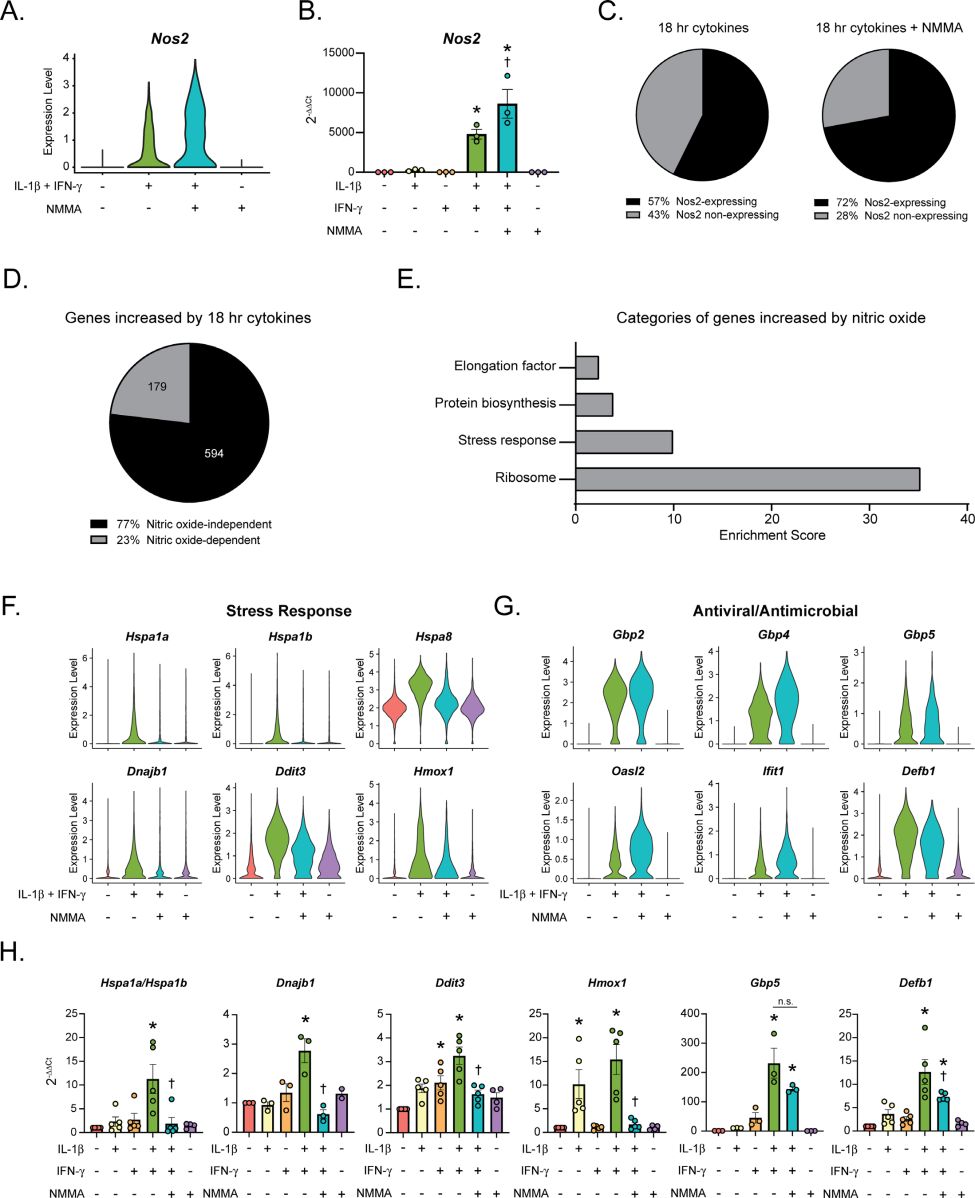
Nitric oxide-dependent gene expression changes in β-cells. (A) Violin plot showing expression level of *Nos2* mRNA in β-cells following indicated cytokine treatments (from scRNA-seq data). (B) *Nos2* mRNA accumulation determined by qRT-PCR in mouse islets treated with the indicated cytokine combinations for 18 h. Error bars represent SEM. Results are from three independent experiments. *P < 0.05 (vs untreated), ^†^*P* < 0.05 (vs. IL-1β + IFN-γ). (C) Pie charts indicating percentages of β-cells expressing *Nos2* mRNA following 18 h treatment with cytokines or with cytokines plus NMMA (from scRNA-seq data). (D) Pie chart showing the percentage of genes increased in β-cells following 18 h cytokine stimulation that are increased in a nitric oxide-dependent manner (from scRNA-seq data). Absolute number of genes are shown in the pie chart. Nitric oxide dependence was determined by differential expression analysis between β-cells treated with cytokines for 18 h and β-cells treated with cytokines combined with NMMA for 18 h. (E) Enriched categories among the genes significantly increased by nitric oxide in β-cells as assessed by DAVID analysis (from scRNA-seq data). (F and G) Violin plots showing the expression level of selected stress response genes (F) or protective genes (G) in β-cells from each of the four treatment groups (from scRNA-seq data). (H) Expression of selected genes (compared to *Gapdh* levels) determined by qRT-PCR using mouse islets treated as indicated for 18 h. Concentrations are as follows: 10 U/mL IL-1β, 150 U/mL IFN-γ, and 2 mM NMMA. Error bars represent SEM. Results are from three to five independent experiments with statistical significance indicated. *P < 0.05 (vs. untreated), ^†^*P* < 0.05 (vs. IL-1β + IFN-γ), n.s. (not significant).

As expected, the gene encoding iNOS (*Nos2*), is significantly increased in β-cells following 18 h exposure to IL-1β and IFN-γ (Figure 3A) ^34^. Nitric oxide inhibits mitochondrial oxidation, thus depleting ATP levels and limiting mRNA transcription ^45^. NOS inhibition by NMMA attenuates the inhibitory effects of nitric oxide on transcription, leading to increased mRNA levels of *Nos2* (Figure 3A). This expression pattern was validated by qRT-PCR to assess *Nos2* mRNA accumulation in mouse islets following cytokine treatment (Figure 3B). Similar to our analysis of acute (6 h) cytokine exposure ^23^, a subset of β-cells were not responsive to cytokines. Only 57% of β-cells increased *Nos2* expression following cytokine treatment, and this percentage increased to 72% after addition of NMMA (Figure 3C). Since β-cells produce micromolar levels of nitric oxide following expression of iNOS ^16–22^, we evaluated the changes in gene expression in response to cytokines that are dependent or independent of nitric oxide. We performed differential expression analysis comparing gene expression in cells treated with cytokines to cells treated with cytokines together with NMMA (Sample 2 vs. Sample 3; Table S3). Genes that were significantly increased in response to cytokines (Sample 2 vs. Sample 1) that were also significantly decreased by the addition of NMMA (Sample 3 vs. Sample 2) were determined to be increased in a nitric oxide-dependent manner. This analysis yielded 179 genes (23% of those increased in response to cytokines) in β-cells (Figure 3D). DAVID analysis of the nitric oxide-dependent genes revealed enriched categories of Ribosome, Stress response, Protein Biosynthesis, and Elongation factor (Figure 3E). This increase in genes associated with translation regulation is likely a compensatory response to inhibition of translation by nitric oxide ^29^. Examples of nitric oxide-dependent genes are those encoding heat shock proteins (like *Hspa8* and *Dnajb1*), *Hmox1*, and *Ddit3*, as expected (Figure 3F) ^41–43,46^. We validated the regulation of selected genes by nitric oxide by qRT-PCR using mouse islets (Figure 3H). *Hspa1a* and *Hspa1b*, which encode subunits of Hsp70, are not significantly decreased by NMMA treatment as assessed by scRNA-seq but are nitric oxide-dependent as assessed by qRT-PCR (Figure 3H). On the other hand, genes encoding antiviral guanylate binding proteins and other immune-associated genes are largely nitric oxide-independent (Figure 3G and H). This is expected, as genes in these categories are increased in β-cells following acute cytokine exposure when nitric oxide levels are low ^19,23^. An exception to this is the antimicrobial gene *Defb1*, which displays nitric oxide-dependent regulation by both scRNA-seq and by qRT-PCR following 18 h cytokine stimulation (Figure 3G and H).

### Factors Controlling β-cell Identity and Function are Decreased Following 18 h Cytokine Treatment

**Figure 4.**
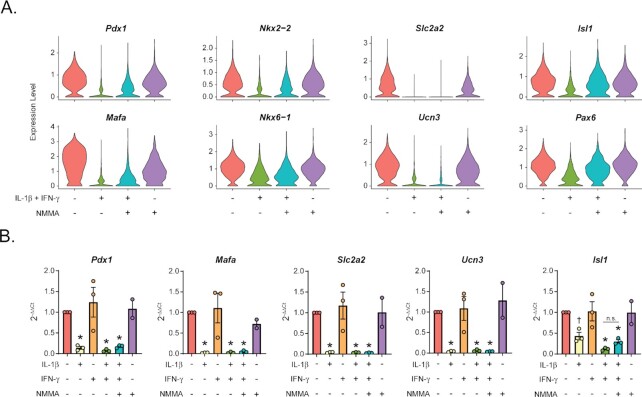
β-cell identity factors are decreased following 18 h cytokine stimulation. (A) Violin plots showing the expression level of selected identity genes in β-cells from each of the four treatment groups. (B) Expression of selected genes (compared to *Gapdh* levels) determined by qRT-PCR using mouse islets treated with the indicated treatments for 18 h. Concentrations are as follows: 10 U/mL IL-1β, 150 U/mL IFN-γ, and 2 mM NMMA. Error bars represent SEM. Results are from three independent experiments with statistical significance indicated. *P < 0.05 (vs. untreated), n.s. (not significant).

Among the genes decreased by cytokines in β-cells are several involved in identity maintenance, including transcription factors (*Pdx1, Mafa, Nkx2-2, Nkx6-1, Isl1*, and *Pax6*), the β-cell glucose transporter (*Slc2a2*), and a gene associated with mature β-cells (*Ucn3*; Figure 4A) ^47–54^. We observed no increase in β-cell “disallowed genes” or other genes associated with β-cell “dedifferentiation” (Figure S3A and B) ^55–57^. Our sequencing analysis revealed that some of these identity genes are partially or completely dependent on nitric oxide (they are decreased in the cytokine-treated sample compared to the untreated sample and are increased in the sample treated with cytokines and NMMA compared to the sample treated with cytokines alone), while others appear to be nitric oxide-independent (Figure 4A). However, qRT-PCR analysis using intact mouse islets suggests that all genes assessed are primarily regulated by IL-1β with minimal contribution from nitric oxide (Figure 4B). In agreement, many of these identity genes were previously observed to be decreased in β-cells following 6 h cytokine exposure ^23^, when nitric oxide levels are low ^19^, again suggesting a primarily nitric oxide-independent regulation. Attenuation of known nitric oxide-dependent genes by the addition of NMMA, including *Hspa1a/Hspa1b* and *Hmox1* (Figure 3H), suggests that this discrepancy is not due to use of an ineffective inhibitor in the qRT-PCR analysis.

### Effects of Cytokines on Gene Expression in Non-β Endocrine Cells

**Figure 5.**
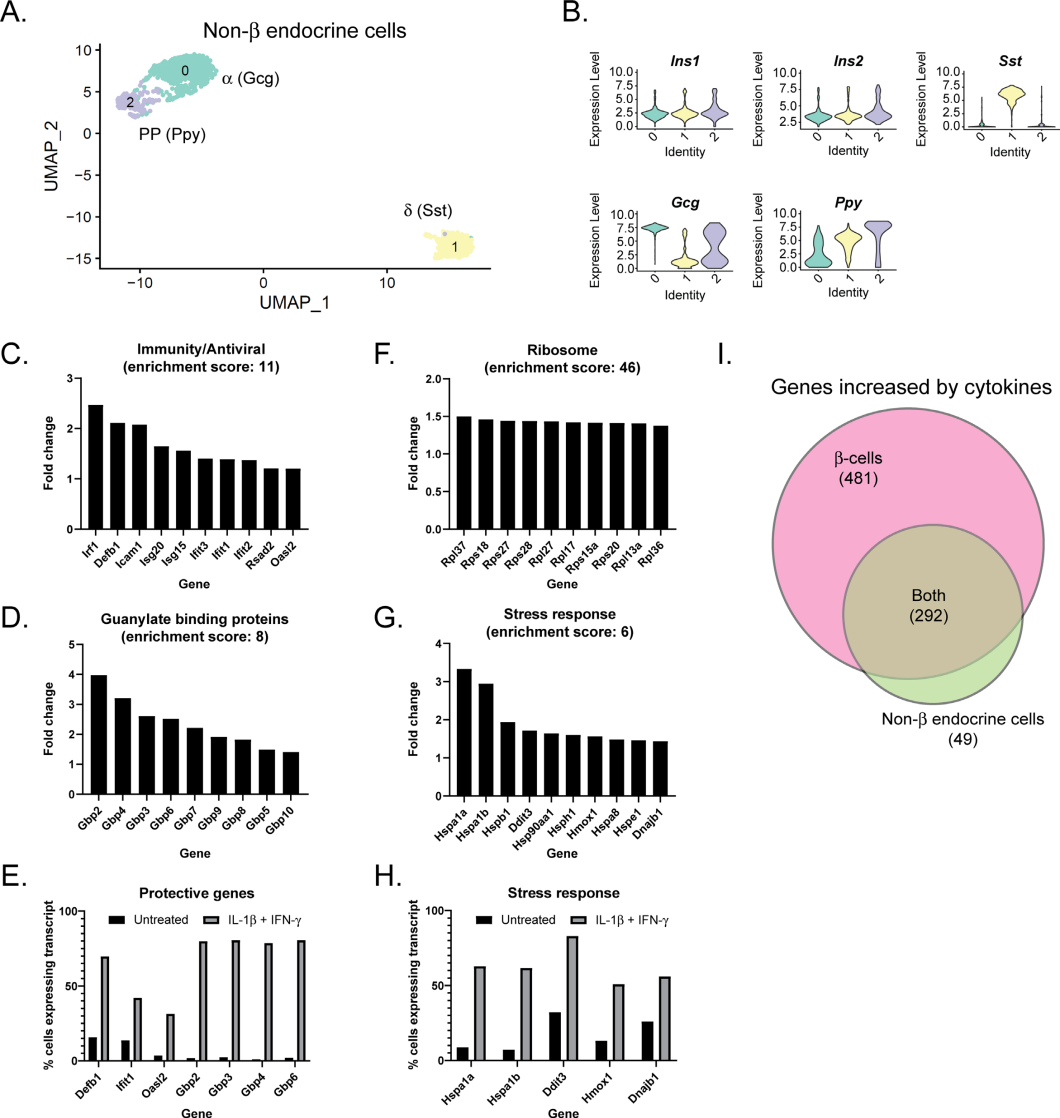
Categories of genes changed in non-β endocrine cells following 18 h cytokine treatment. (A–B) α-, δ-, and PP-cells were computationally isolated from the complete dataset and reclustered. A total of 1069 cells (373 untreated, 159 treated with cytokines, 263 treated with cytokines together with NMMA, and 274 treated with NMMA alone) were used in this analysis. UMAP plot depicting clusters of non-β endocrine cells. Cell identity was assigned based on enrichment for genes indicated (A). Violin plots indicating expression levels of *Ins1, Ins2, Gcg, Sst*, and *Ppy* mRNA in each of the three clusters (B). (C, D, F, and G) Genes that were significantly different in non-β endocrine cells following 18 h cytokine stimulation compared to untreated cells were subjected to functional annotation clustering using the Database for Annotation, Visualization and Integrated Discovery (DAVID). Fold change (over untreated) of representative genes in the selected enriched categories of Immunity/antiviral (C), Guanylate binding protein (D), Ribosome (F), and Stress response (G) are shown. (E and H) Percent of non-β endocrine cells expressing selected genes involved in protection (E) or stress response (H) in the untreated or cytokine-treated conditions. (I) Venn diagram comparing the genes increased in β-cells following 18 h cytokine treatment compared to those also increased or decreased in non-β endocrine cells.

We recently demonstrated that non-β endocrine cells (α-, δ-, and PP-cells) respond to acute (6 h) cytokine exposure in a manner very similar to β-cells ^23^. To better understand how non-β endocrine cells respond to 18 h cytokine exposure, we computationally combined Clusters 2 and 4 (α- and δ-cells, respectively) from the total dataset and analysed these populations following reclustering. Hormone gene expression in this subpopulation revealed a small cluster of contaminating β-cells. After removing those insulin-expressing cells, we were left with 1069 cells divided into three clusters of non-β endocrine cells enriched for α-, δ-, and PP-cells based on hormone gene expression (Figure 5A and B). Similarly to our original clustering analysis (Figure 1B and C), a pure *Ppy*-expressing population was difficult to identify, as the *Ppy*-enriched cluster also expressed *Gcg* (Figure 5B). We then performed differential expression analysis to determine genes significantly changed in response to each treatment (compared to the untreated sample; Table S4). We additionally performed differential expression analysis for α- and δ-cells individually (Tables S5 and S6), but found that, in general, the statistical power was greater when analysing all non-β endocrine cells as one group due to the relatively low number of non-β endocrine cells in the islets. In total, 881 genes (341 increased and 540 decreased) were significantly changed in non-β endocrine cells in response to 18 h cytokine stimulation compared to no cytokine stimulation. Similar to our analysis with the β-cell population, we used DAVID to identify enriched categories of genes changed by cytokine treatment. As expected, guanylate binding proteins and other antiviral or immune-associated genes were increased in non-β endocrine cells following 18 h cytokine stimulation (Figure 5C and D). These genes, as in β-cells, are highly inducible, expressed in fewer than 20% of cells basally but increased to greater than 60% following cytokine stimulation in several cases (Figure 5E). Many of these genes were increased in α- and δ-cells after acute cytokine exposure ^23^. Mirroring the β-cell population, nearly 20% of the genes increased in non-β endocrine cells following 18 h cytokine stimulation fell into the category of ribosomal proteins (Figure 5F). Genes associated with a cellular stress response (including a number of heat shock proteins, *Ddit3*, and *Hmox1*) are also increased, not only in terms of expression level, but also in terms of the percentage of cells expressing the transcripts (Figure 5G and H). These results suggest that both β- and non-β endocrine cells respond in a similar manner to cytokine stimulation. Indeed, of the 341 genes increased in non-β endocrine cells following 18 h cytokine exposure, 292 of them (86%) are also increased in β-cells (Figure 5I).

### Non-β Endocrine Cells Express iNOS in Response to Cytokines

**Figure 6.**
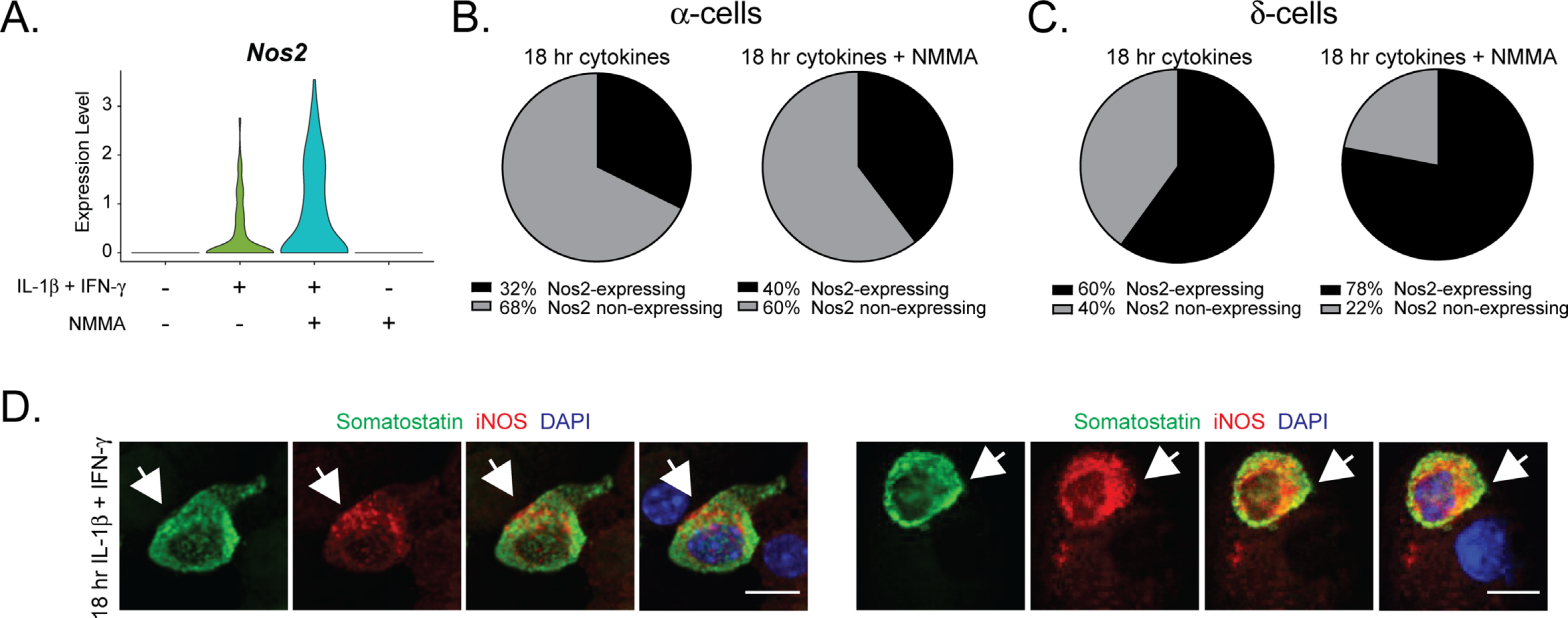
iNOS expression in non-β endocrine cells. (A) Violin plot showing expression level of *Nos2* mRNA in non-β endocrine cells following indicated cytokine treatments (from scRNA-seq data). (B and C) Pie charts indicating percentages of α-cells (B) or δ-cells (C) expressing *Nos2* mRNA following 18 h treatment with cytokines or with cytokines plus NMMA (from scRNA-seq data). (D) Immunofluorescence images showing examples of dissociated mouse islet cells with co-localization of somatostatin and iNOS protein following 18 h cytokine treatment. Arrowheads indicate somatostatin-containing cells also containing iNOS. Scalebar = 10 μm.

Although β-cells are thought to be the islet endocrine source of cytokine-derived nitric oxide ^22,34,35^, we have previously observed *Nos2* gene expression in a subset of α-, δ-, ad PP-cells after acute cytokine treatment ^23^. Consistently, *Nos2* mRNA is increased in non-β endocrine cells following 18 h cytokine treatment and is further augmented with the addition of NMMA (Figure 6A). Specifically, 32% of α-cells and 60% of δ-cells express *Nos2* in response to cytokines, and these percentages increase to 40% and 78%, respectively, in the presence of NMMA (Figure 6B and C). In agreement with our previous studies, β- and δ-cells appear to be more responsive to cytokines than α-cells, as greater subpopulations of these two cell types express detectable levels of cytokine-stimulated *Nos2* mRNA ^23^. Further, we observed colocalization of somatostatin with iNOS by immunofluorescence microscopy in dissociated mouse islet cells following 18 h stimulation with IL-1β and IFN-γ (Figure 6D). To our knowledge, this is the first demonstration of cytokine-stimulated iNOS protein expression in islet non-β endocrine cells. Colocalization of iNOS with glucagon in dissociated mouse islet cells is limited, likely because the levels of iNOS in glucagon-containing cells appear to be only slightly above the background level, although some examples can be found (Figure S4). This observation is consistent with the lower percentage of α-cells that increase *Nos2* expression following cytokine treatment, as compared to the percentage of β- and δ-cells (Figures 3C, 6B and C). Together, these results suggest that islet endocrine cells other than β-cells express iNOS mRNA and protein in response to cytokine exposure.

### Nitric Oxide-Dependent Gene Expression Changes in Non-β Endocrine Cells

**Figure 7.**
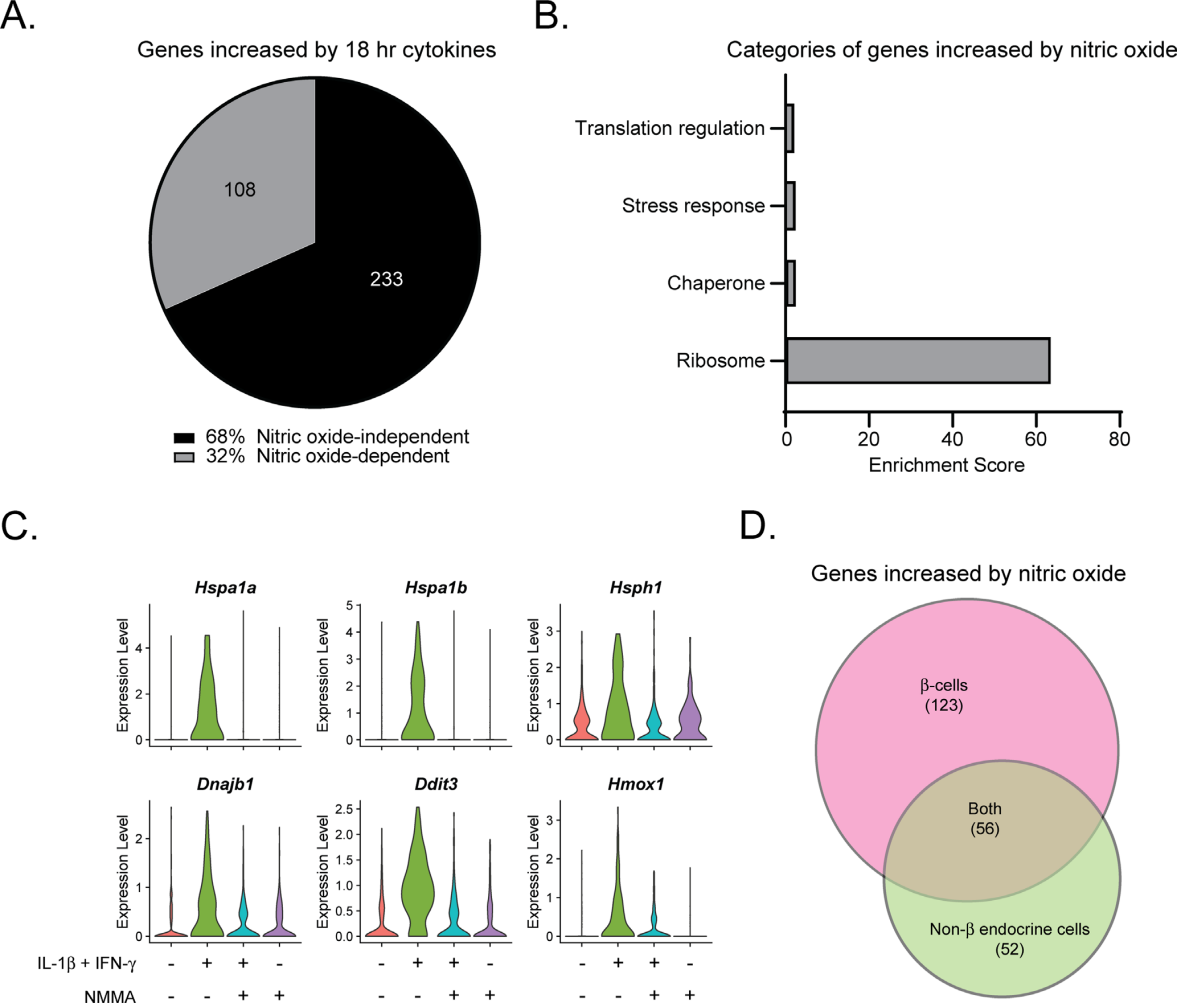
Nitric oxide-dependent gene expression changes in non-β endocrine cells. (A) Pie chart showing the percentage of genes increased in non-β endocrine cells following 18 h cytokine stimulation that are increased in a nitric oxide-dependent manner. Absolute number of genes are shown in the pie chart. (B) Enriched categories among the genes significantly increased by nitric oxide in non-β endocrine cells as assessed by DAVID analysis. (C) Violin plots showing the expression level of selected genes in non-β endocrine cells from each of the four treatment groups. (D) Venn diagram comparing genes increased in β-cells by nitric oxide to those increased in non-β endocrine cells by nitric oxide.

Since islet non-β endocrine cells appear to express *Nos2* mRNA and iNOS protein following cytokine exposure, we hypothesized that they may also display nitric oxide-dependent gene expression changes. Indeed, of the 341 genes significantly increased in response to cytokines in non-β endocrine cells, 108, or approximately 32%, were increased in a nitric oxide-dependent manner (Figure 7A). DAVID analysis of the nitric oxide-dependent genes revealed enriched categories of Ribosome, Chaperone, Stress response, and Translation regulation (Figure 7B). Expression patterns of heat shock proteins (*Hspa1a, Hspa1b, Hsph1*, and *Dnajb1*), heme oxygenase 1 (*Hmox1*), and Chop (*Ddit3*) are nearly identical to the expression patterns observed in β-cells (Figures 7C and 3F). In fact, of the 108 genes increased in non-β endocrine cells in a nitric oxide-dependent manner, 56 genes (52%) are also increased in β-cells in a nitric oxide-dependent manner (Figure 7D) suggesting that all islet endocrine cells experience nitric oxide signaling in response to cytokine stimulation. However, assessment of the expression pattern of these genes in non-endocrine cells captured in our dataset reveals a lack of nitric oxide dependent regulation (Figure S5), suggesting that nitric oxide signaling in response to cytokine exposure is selective for islet endocrine cells.

### Genes Controlling Non-β Endocrine Cell Identity and Function are Decreased Following 18 h Cytokine Treatment

**Figure 8.**
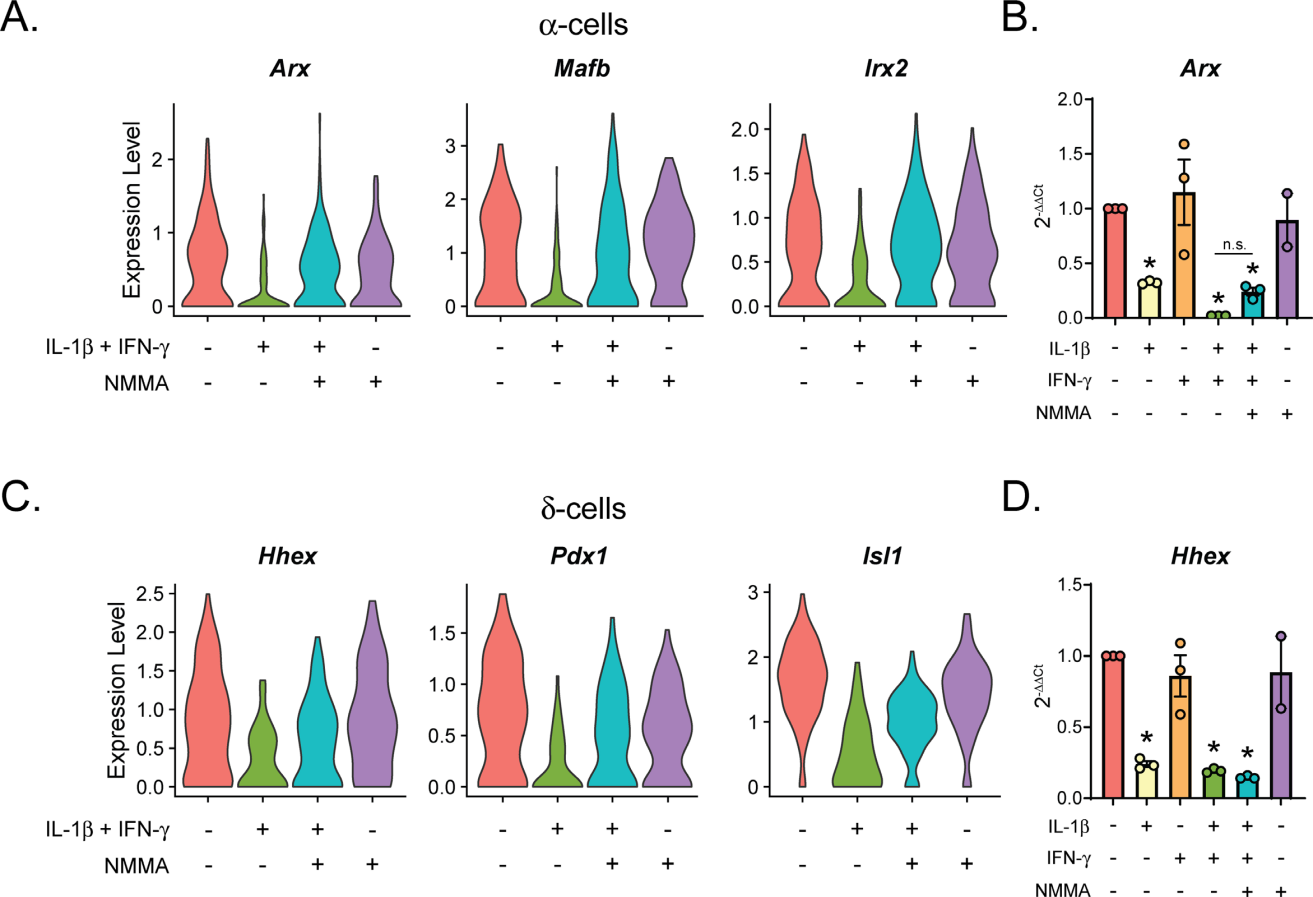
Non-β endocrine cell identity factors are decreased following 18 h cytokine stimulation. (A and C) Violin plots showing the expression level of selected identity genes in α-cells (A) or δ-cells (C) from each of the four treatment groups. (B and D) mRNA accumulation of *Arx* (B) or *Hhex* (D) determined by qRT-PCR using mouse islets treated with the indicated cytokine combinations for 18 h. Concentrations are as follows: 10 U/mL IL-1β, 150 U/mL IFN-γ, and 2 mM NMMA. Error bars represent SEM. Results are from three independent experiments with statistical significance indicated. *P < 0.05 (vs. untreated), n.s. (not significant).

Because we observed decreased expression of several genes involved in β-cell identity maintenance after 18 h cytokine exposure (Figure 4), we hypothesized that the same will be true for non-β endocrine cells. Indeed, *Arx, Mafb*, and *Irx2*, transcription factors regulating α-cell specification and glucagon expression, are decreased in the α-cell population in response to 18 h stimulation with IL-1β and IFN-γ (Figure 8A) ^58–60^. Similar to the β-cell identity factors, these α-cell identity factors appeared to be regulated by nitric oxide when assessed by scRNA-seq. However, qRT-PCR analysis of *Arx* in mouse islets suggests minimal regulation of this gene by nitric oxide (Figure 8B). This is consistent with our previous study suggesting that repression of islet identity factors by IL-1β is nitric oxide-independent ^23^. *Hhex, Pdx1*, and *Isl1*, transcription factors controlling δ-cell specification and identity maintenance, are decreased in the δ-cell population following 18 h cytokine treatment (Figure 8C) ^60–63^. Again, scRNA-seq analysis suggests nitric oxide-dependent repression of these identity factors, but qRT-PCR analysis of *Hhex* using mouse islets suggests nitric oxide-independent regulation (Figure 8D). Together with data presented in Figure 4, these results suggest that cytokines repress identity factors of non-β islet endocrine cells in a manner that is primarily nitric oxide-independent.

### Cytokine Signaling is Negatively Correlated With Heat Shock Protein Gene Expression

**Figure 9.**
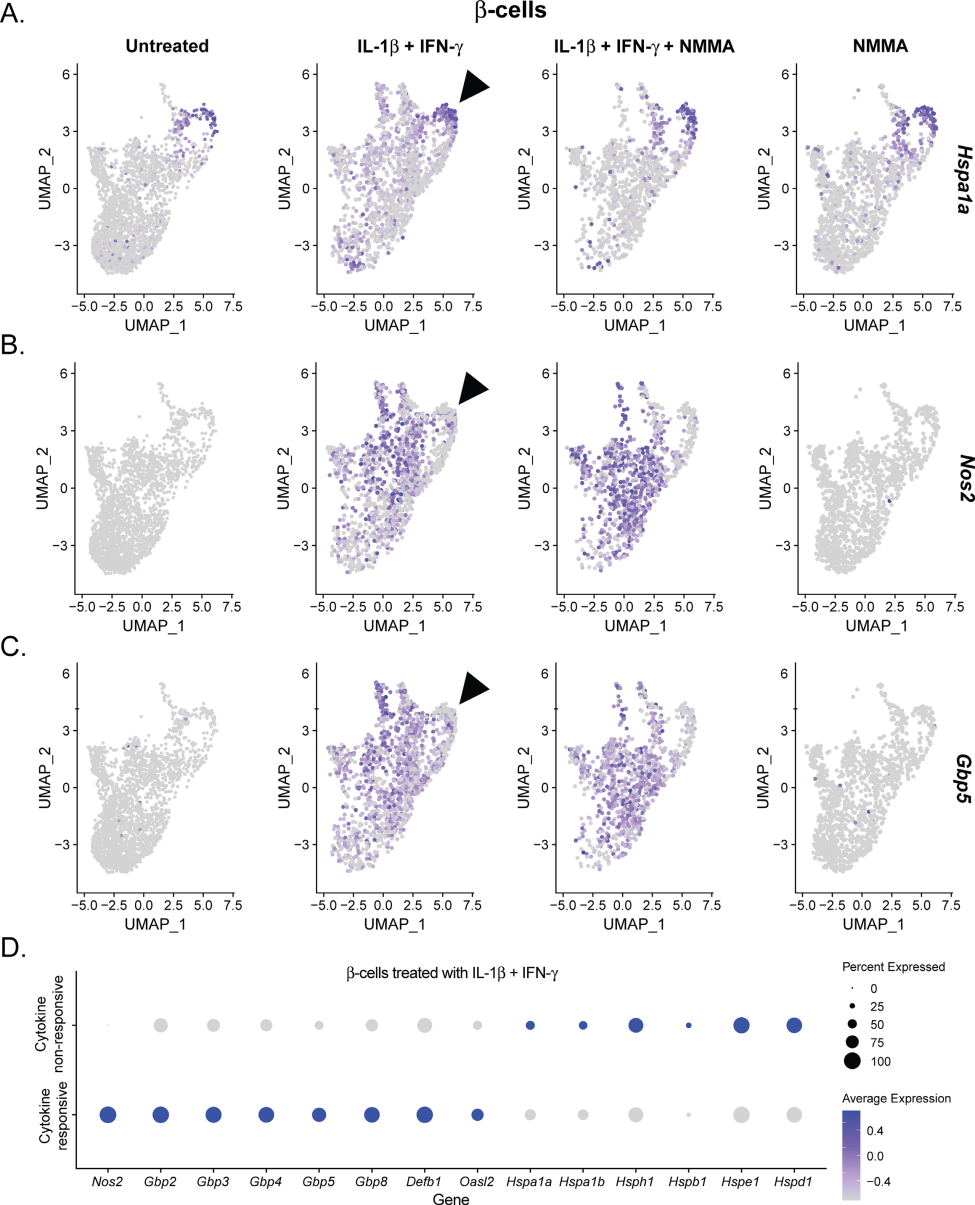
Cellular stress is negatively associated with cytokine signaling. (A–C) UMAP plots segregated by sample and colored to indicate expression level of *Hspa1a* (A), *Nos2* (B), and *Gbp5* (C) in β-cell clusters. Arrowhead points out the population of cells with high expression of *Hspa1a* that fails to induce *Nos2* and *Gbp5* in response to 18 stimulation with IL-1β and IFN-γ. (D) Dot plot depicting enrichment of selected genes between cytokine responsive (*Nos2*-expressing) and cytokine non-responsive (*Nos2* non-expressing) β-cells treated with IL-1β and IFN-γ for 18 h.

As shown in Figure 3, only 57% of β-cells express detectable levels of iNOS mRNA (*Nos2*) following 18 h cytokine simulation. Even with the addition of NMMA, only 72% of β-cells express *Nos2*, leading us to hypothesize that there may be a distinguishing feature of the remaining 28%, preventing them from responding to cytokines. Analysis of the expression pattern of *Nos2* across the β-cell population reveals a subpopulation with high expression of *Hspa1a* that failed to increase *Nos2* mRNA in response to 18 h exposure to IL-1β and IFN-γ or to the combination of the two cytokines with NMMA (Figure 9A and B). Because this subpopulation persisted across all treatment groups and both biological replicates, it is likely that these cells were stressed prior to the cytokine treatment, leading to elevated levels of heat shock proteins. In addition to *Nos2*, β-cells with elevated levels of *Hspa1a* also failed to increase expression of antiviral and other immune-associated genes, including *Gbp5* (Figure 9C). This observation is consistent with our previous scRNA-seq study in which we found a similar subpopulation of β-cells enriched for heat shock proteins that failed to increase *Nos2* expression after 6 h cytokine stimulation and aligns with reports that heat shock inhibits cytokine-stimulated iNOS expression is islets ^23,46,64,65^. To strengthen our conclusion that cellular stress is negatively correlated with cytokine signaling, we performed differential expression analysis between “cytokine responsive” β-cells (those that expressed *Nos2*) and “cytokine non-responsive” β-cells (those that did not express *Nos2*) in Sample 2 (18 h exposure to IL-1β and IFN-γ). Cytokine responsive β-cells were enriched for antiviral and antimicrobial genes (such as guanylate binding proteins, *Defb1*, and *Oasl2*) while cytokine non-responsive β-cells were enriched for several heat shock proteins (Figure 9D). The same pattern of gene enrichment was observed using cytokine responsive and non-responsive β-cells from Sample 3 (18 h exposure to cytokines + NMMA), which avoided the potential confounding effects of nitric oxide-stimulated heat shock gene expression (Figure S6A). These findings provide additional support for our hypothesis that cytokine non-responsive cells were stressed prior to cytokine exposure. Finally, comparison of *Hspa1a*-expressing to *Hspa1a* non-expressing β-cells following cytokine exposure and NMMA (Sample 3) revealed a negative correlation to cytokine signaling. *Hspa1a*-expressing β-cells, while expressing other heat shock proteins, failed to express antiviral and antimicrobial genes following cytokine stimulation (Figure S6B). Together, these results suggest that induction of cellular stress attenuates cytokine signaling in β-cells.

## Discussion

Type 1 diabetes is an autoimmune disease caused by selective destruction of β-cells. Nitric oxide is produced in β-cells in response to proinflammatory cytokines like IL-1β and IFN-γ and inhibits mitochondrial oxidative metabolism and insulin secretion, with prolonged exposure causing cell death ^16–19,35,66,67^. Because of these observations, cytokines and cytokine-derived nitric oxide are widely thought to be damaging to β-cells and have been suggested as mediators of T1D development ^68^. However, there are logical arguments to support a physiological role for IL-1β signaling in β-cells, particularly regarding nitric oxide production. In support of this view, there are large increases in serum IL-1β levels in response to an acute infection ^69^, and consistent with their glucose sensing role, islets are highly vascularized and receive the majority of the pancreatic blood flow ^70,71^. This vascular architecture is essential for the control of glucose homeostasis, but also leads to β-cells being bathed in IL-1β during an infection. Furthermore, β-cells have limited capacity to proliferate ^72^. Therefore, if cytokines were solely damaging to β-cells, we might expect a higher incidence of T1D than currently observed. In support of this hypothesis, cytokines stimulate protective gene expression in β-cells ^23^, and, through production of nitric oxide, protect β-cells from apoptosis ^26^ and viral infection ^24,25^.

**Figure 10.**
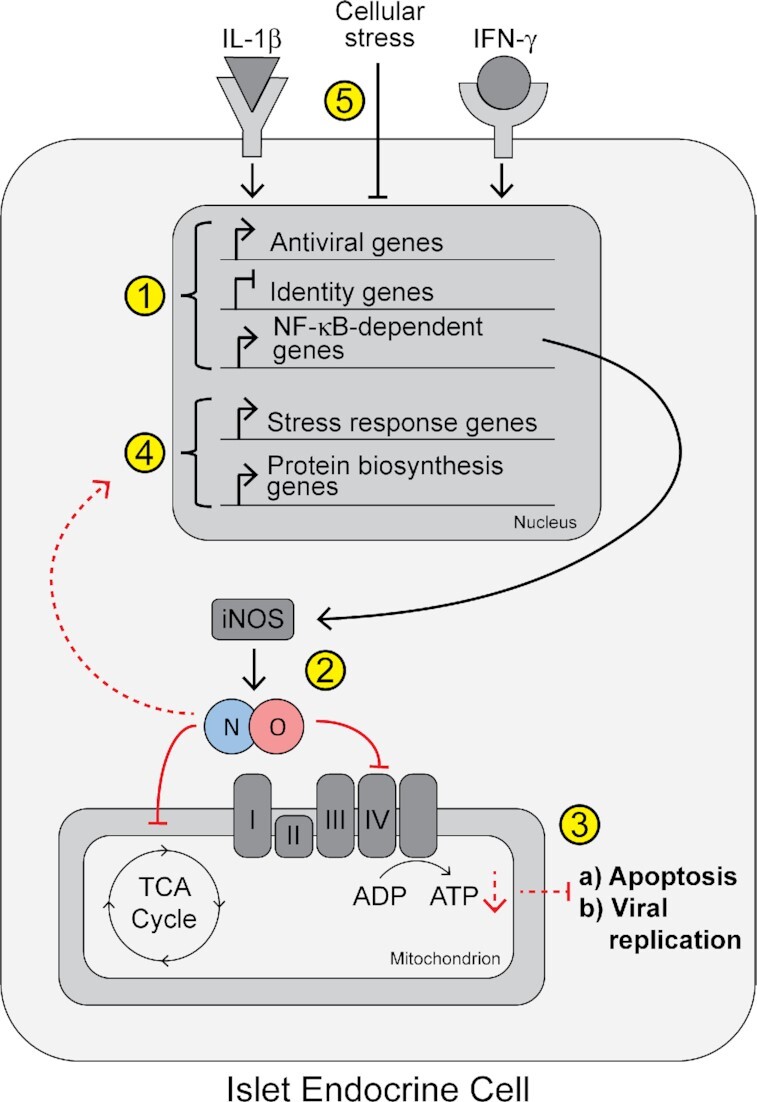
Model. (1) Exposure to IL-1β and IFN-γ causes islet endocrine cells (β-, α-, and δ-cells) to increase expression of antiviral/antimicrobial genes and NF-κB-dependent genes, including *Nos2*, and to decrease expression of identity factors. (2) *Nos2* gene expression leads to iNOS protein expression and generation of micromolar levels of nitric oxide, which directly inhibits complex IV of the electron transport chain and aconitase of the TCA cycle, indirectly leading to decreased intracellular ATP levels. (3) Inhibition of mitochondrial oxidation by nitric oxide attenuates apoptosis and viral replication. (4) Nitric oxide indirectly causes increased expression of genes involved in stress response, including heat shock proteins, *Hmox1*, and *Ddit3*, and increased expression of genes involved in protein biosynthesis. (5) Cellular stress, such as heat shock or endoplasmic reticulum stress, attenuates IL-1β and IFN-γ signaling pathways. Solid red lines represent direct actions of nitric oxide. Dashed red lines represent indirect actions of nitric oxide.

We previously demonstrated that β-, α-, and δ-cells respond in a similar manner to acute (6 h) exposure to IL-1β and IFN-γ ^23^. The same trend was observed here with a longer cytokine exposure (18 h), with 86% of the genes increased in non-β endocrine cells (a subpopulation of our dataset that included α-, δ-, and PP-cells) also increased in β-cells (Figure 5I). Of these cytokine-induced genes, 77% were increased in a nitric oxide-independent manner in β-cells and 68% in non-β endocrine cells (Figures 3D and 7A). Among the genes regulated in this way are antiviral (including a number of guanylate-binding proteins), antimicrobial, and other immune-associated genes (Figures 3, 7 and   10). This is consistent with our previous observation that acute cytokine exposure, before nitric oxide accumulates, increases a subset of antiviral and other protective genes in all islet endocrine cells and that several of these genes are stimulated by IL-1β ^19,23^. Importantly, we did not observe an increase in expression of pro-apoptotic genes in β-cells, neither in our previous study after 6 h cytokine exposure nor in the current study following 18 h stimulation (Figure S2) ^23^, further suggesting that the primary response of islet endocrine cells to inflammatory cytokines is protective and likely not the induction of apoptosis.

Another category of genes regulated in a primarily nitric oxide-independent manner is islet identity factors, including genes critical for maintaining β-cell function, like *Pdx1, Mafa*, and *Slc2a2* (Figure 4); genes controlling α-cell identity maintenance, like *Arx, Mafb*, and *Irx2* (Figure 8A); and genes regulating δ-cell identity maintenance, like *Hhex, Pdx1*, and *Isl1* (Figure 8C), all of which were decreased following 18 h cytokine exposure (Figure 10). Specifically, selected genes assessed by qRT-PCR were decreased by IL-1β but not by IFN-γ (Figures 4B, 8B and D), consistent with previous studies ^23,73,74^. Repression of these identity factors did not coincide with increased expression of “disallowed” genes or of other genes associated with “dedifferentiation” (Figure S3) ^55–57^ indicating that these cells have not lost their cellular identity and that the repression may be reversible under these conditions. Decreased expression of identity factors may be an additional mechanism by which cytokines protect β-cells by directing cellular energy away from glucose sensing and insulin secretion and toward protective pathways under conditions of environmental threats. Some of these identity factors appeared to be decreased in a nitric oxide-dependent manner when assessed by RNA-seq, but not when assessed by qRT-PCR (Figures 4 and 8). This discrepancy may be attributed to differences in the technique or in the cell populations used, as scRNA-seq was performed using individual cells clustered by cell type, while qRT-PCR was performed using islets, which contain a heterogeneous cell population. Since identity factors were similarly repressed after acute cytokine treatment when nitric oxide levels are low^23^, it is likely that the primary mechanism by which IL-1β represses these genes is nitric oxide-independent. However, nitric oxide may provide additional repression of cellular identity by a secondary mechanism, such as depletion of ATP, which may negatively impact gene transcription ^45^. Indeed, augmentation of some antiviral genes by addition of the NOS inhibitor supports this hypothesis (Figure 3G).

Because our recent scRNA-seq study was performed following acute (6 h) cytokine treatment, when nitric oxide levels are low^19^, we previously focused on early islet cell responses to cytokines that were largely nitric oxide independent ^23^. In the current study, we focused on identifying nitric oxide-dependent changes following 18 h cytokine exposure by utilizing a nitric oxide synthase inhibitor, NMMA. Crucially, this length of exposure allowed us to identify nitric oxide dependent changes occurring before nitric oxide-mediated damage becomes irreversible ^29,30^. While most genes increased by cytokines were not regulated by nitric oxide in a statistically significant manner, we observed high enrichment for categories of genes regulated by nitric oxide, including ribosomal proteins, genes involved in cellular stress responses, and genes involved in protein biosynthesis, in both β-cells and non-β endocrine cells (Figures 3, 7 and   10). Genes of interest included heat shock proteins *Hspa1a, Hspa1b, Hspa8*, and *Dnajb1*; the ER-stress associated gene *Ddit3*; and the antioxidant gene *Hmox1*. It is likely that ribosomal proteins and other genes involved in protein biosynthesis are stimulated as a compensatory response to inhibition of protein synthesis by nitric oxide ^29^. While it is possible that nitric oxide negatively affects translation efficiency of antiviral genes into proteins, we have previously shown that these same antiviral genes are stimulated by acute cytokine treatment, likely before nitric oxide inhibits protein biosynthesis ^23^. Temporally, it is likely that the antiviral genes, which are stimulated as an early cytokine response, are translated into proteins before nitric oxide accumulation affects protein translation.

Because β-cells are thought to be the only islet endocrine cell type that produces nitric oxide in response to IL-1β stimulation ^22,34,35^, we were surprised to observe nitric oxide-dependent regulation of many of the same genes in non-β endocrine cells as in the β-cell population (Figure 7C). Of the 108 genes significantly increased by nitric oxide in non-β endocrine cells, over half were also found to be nitric oxide-dependent in β-cells (Figure 7D). Interestingly, nitric oxide-dependent regulation of these genes was not observed in the non-endocrine cells (eg, endothelial cells, macrophages, and mesenchymal cells) captured in our analysis (Figure S5), suggesting that the effects are likely due to nitric oxide produced endogenously and not from nitric oxide diffusion from a source to a target cell. If diffusion caused changes in gene expression, it would be expected that nitric oxide would change gene expression in non-endocrine cells. Additionally, subpopulations of α- and δ-cells increased *Nos2* mRNA in response to treatment with IL-1β and IFN-γ (Figure 6B and C), consistent with our previous observation ^23^, and examples of dissociated islet cells co-expressing iNOS and somatostatin were found following 18 h cytokine stimulation (Figure 6D). Our previous studies were unable to detect nitrite accumulation from FACS-purified rat islet non β-cells after stimulation with IL-1β and IFN-γ ^15^. Therefore, it is yet to be determined if the nitric oxide-dependent gene expression changes observed in mouse non-β endocrine cells are cell-autonomous or if they are driven by diffusion of β-cell-derived nitric oxide. Regardless of the source of the nitric oxide, our results suggest that all islet endocrine cells respond to cytokine stimulation by increasing expression of *Nos2* and other immune-associated genes and experience similar nitric oxide-dependent gene expression changes.

Although populations of β-, α-, and δ-cells increased *Nos2* mRNA following cytokine treatment, the response was heterogeneous. *Nos2* was significantly increased in 57% of β-cells, 32% of α-cells, and 60% of δ-cells in response to IL-1β and IFN-γ (Figures 3C, 6B and C). While these percentages increased to 72%, 40%, and 78%, respectively, when NMMA was added, the response was still heterogeneous. We do not think this heterogeneity can be attributed to differences in expression of cytokine receptors. The mRNA levels of the interleukin 1 signaling receptor, type 1 (*Il1r1*) and the first subunit of the IFN-γ receptor (*Ifngr1*) were not different between these two populations of β-cells and were expressed in a similar percentage of cells in both populations (Table S3). The second subunit of the IFN-γ receptor, *Ifngr2*, is increased in the *Nos2*-expressing β-cell population and is expressed in a higher percentage of cells (48% of *Nos2*-expressing cells vs. 32% of *Nos2* non-expressing cells, Table S3). However, this is likely a consequence of a positive feedback loop in which expression of the receptor is increased in response to the cytokine, as *Ifngr2* is among the genes significantly increased by 18 h cytokine treatment (Table S3). We do not think heterogeneity in cytokine responsiveness can be attributed to differences in the expression of *Ifngr2* because several IL-1β-dependent genes, including *Icam1, Sod2*, and *Defb1* are not increased in “cytokine non-responsive” cells, suggesting a failure to respond to either IL-1β or IFN-γ.

The β-cells that failed to express *Nos2* in response to both cytokines corresponded with a subpopulation of cells with high expression of *Hspa1a* and other heat shock proteins (Figure 9A, B and D). This same subpopulation of cells also failed to increase expression of antiviral and other immune-associated genes that are regulated by IFN signaling (Figure 9C and D), suggesting that the inhibition was not limited to IL-1-driven responses, but affected cytokine signaling more broadly, consistent with previous studies ^64,75^. Because this subpopulation persisted across all treatment conditions and both biological replicates, it is likely that these cells were stressed before cytokine exposure, perhaps due to the islet isolation process. Regardless of the source, this finding shows that cellular stress is negatively correlated with cytokine signaling and aligns with previous studies establishing that cytokines fail to stimulate iNOS expression in rodent and human islets under conditions of heat shock or ER stress (Figure 10) ^46,64,65^. We observed a similar subpopulation in our previous scRNA-seq analysis with high expression of heat shock proteins that failed to increase *Nos2* mRNA following acute exposure to IL-1β and IFN-γ ^23^. Interestingly, this cellular stress response is common following human islet isolations ^76^ and may explain the suggestions that human islets do not respond to cytokines in the same manner as rodent islets ^77,78^. Researchers utilizing human islets should assess HSP70 expression levels when making conclusions regarding islet responses to cytokines, as cellular stress may affect experimental outcomes. HSP70 expression itself does not mediate the inhibitory effects on cytokine signaling and, thus should only be used as an index for cellular stress ^75^.

Cytokines and nitric oxide are primarily viewed as damaging to β-cells, but consideration of recent studies suggests that there is a physiological role of IL-1β signaling in β-cells that is designed to inhibit oxidative phosphorylation by nitric oxide. While this inhibition limits insulin secretion, we believe that it does not initially damage the cells but protects them from environmental threats. Indeed, inhibition of mitochondrial metabolism by nitric oxide inhibits DNA damage-mediated apoptosis and prevents viral replication (Figure 10) ^24–26^. While it is true that nitric oxide stimulates genes associated with a cellular stress response that may be interpreted as damaging (Figure 3), ours and others’ observations that cellular stress is negatively correlated with cytokine signaling ^23,46,64,65^, and that cytokine-induced nitric oxide promotes heat shock protein expression in islets ^79^ suggest that nitric oxide may provide an additional layer of protection by providing an internal “off switch” to prevent cytokine-mediated damage after protective signaling has been initiated (Figure 9). This argument is made stronger when remembering that cytokine-mediated damage is reversible, and only after prolonged cytokine stimulation is the damage permanent ^27–29^. The observation made here that nitric oxide affects gene expression in α- and δ-cells suggests that nitric oxide may play a protective role in all islet endocrine cells, not only β-cells.

### Data Availability Statement

Sequencing data from this publication have been deposited in NCBI GEO database under accession number GSE183010.

## Supplementary Material

zqab063_Supplemental_Files
