# Supplementary material for: Cytokine and Nitric Oxide-Dependent Gene Regulation in Islet Endocrine and Nonendocrine Cells
Source: Function (Oxf). 2021 Dec 1;3(1):zqab063. doi: 10.1093/function/zqab063 (PMC8674205; doi:10.1093/function/zqab063)
Supplement: zqab063_Supplemental_Files [file zqab063_supplemental_files.zip › Supplemental materials_zqab063.pdf]

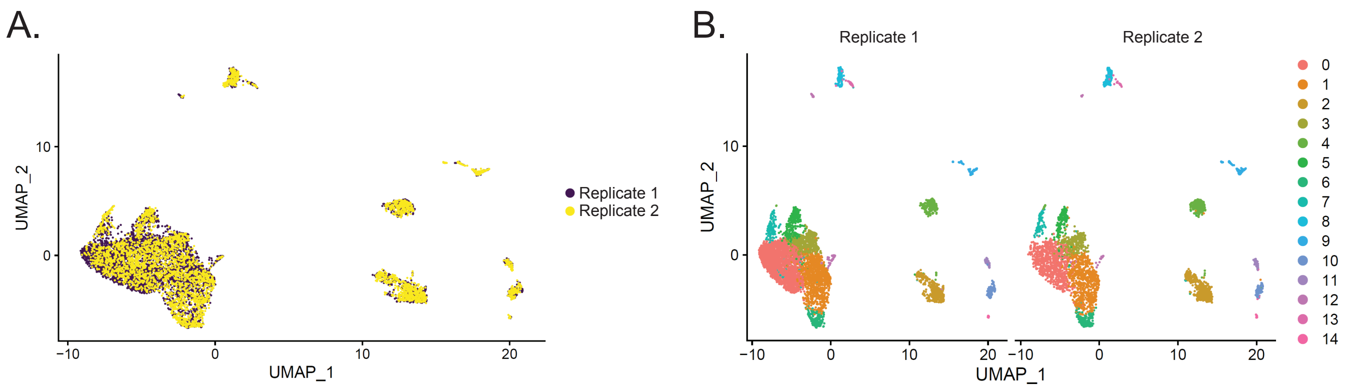

Figure S1: Distribution of two independent scRNA-seq replicates across cell clusters. (A-B) UMAP plots depicting clusters of cells from both scRNA-seq experimental replicates either color coded to indicate replicate of origin (A) or split to indicate the contribution of the replicates to each cluster (B).

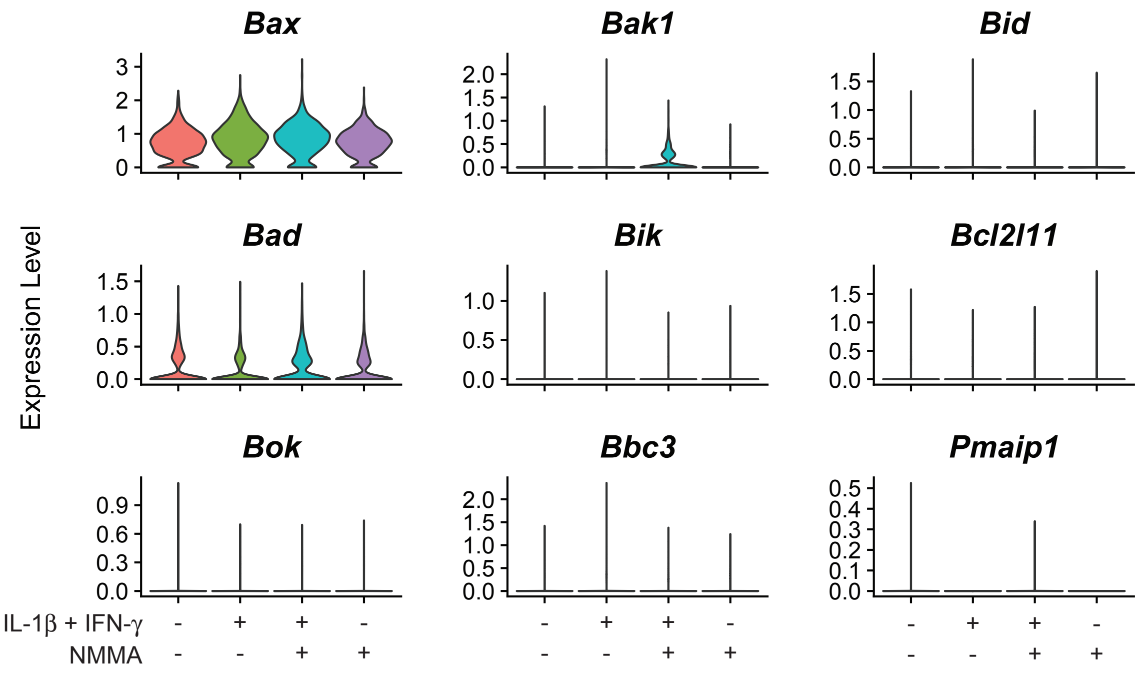

Figure S2: Expression of pro-apoptotic genes in β-cells. Violin plots showing the expression levels of selected pro-apoptotic genes in β-cells in each of the four treatments. Concentrations are as follows: 10 U/mL IL-1β, 150 U/mL IFN-γ, 2 mM NMMA**.**

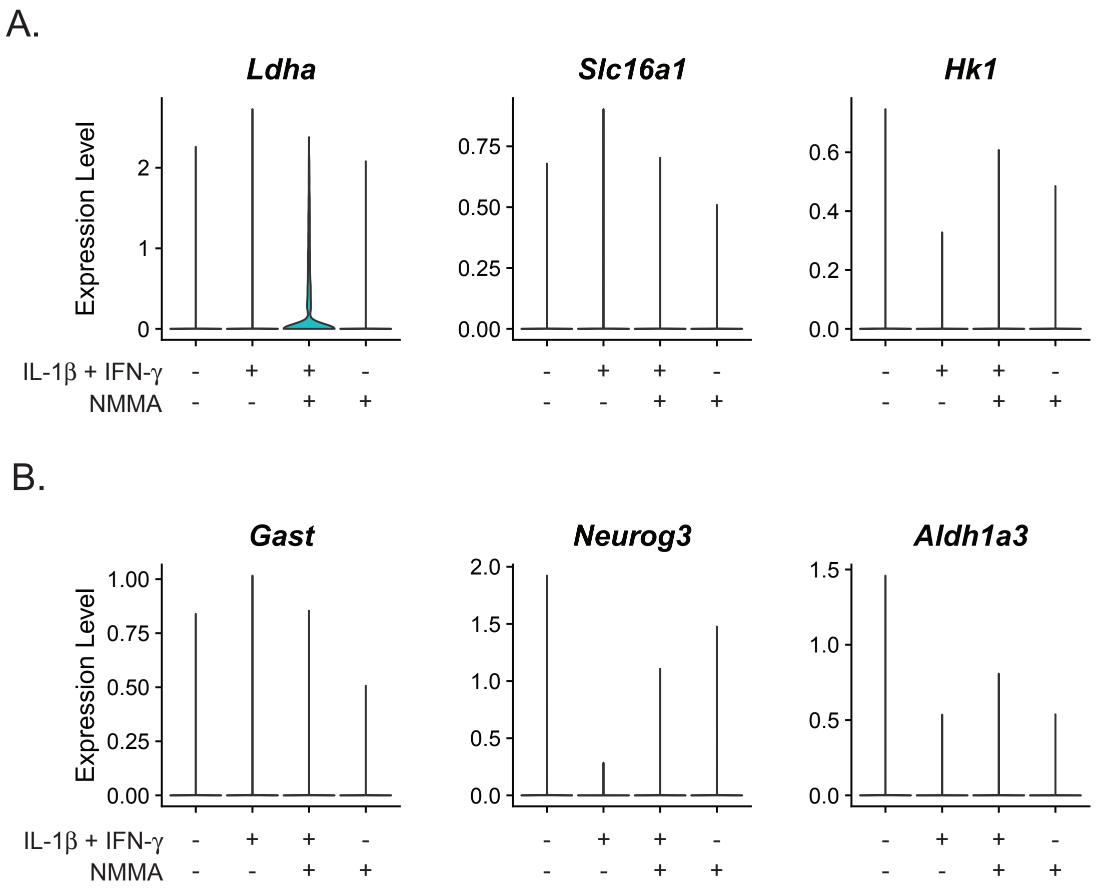

Figure S3: Expression of genes associated with dedifferentiation in β-cells. (A, B) Violin plots showing the expression level of disallowed genes (A) and of dedifferentiation genes (B) in β-cells in each of the four treatments. Concentrations are as follows: 10 U/mL IL-1β, 150 U/mL IFN-γ, 2 mM NMMA.

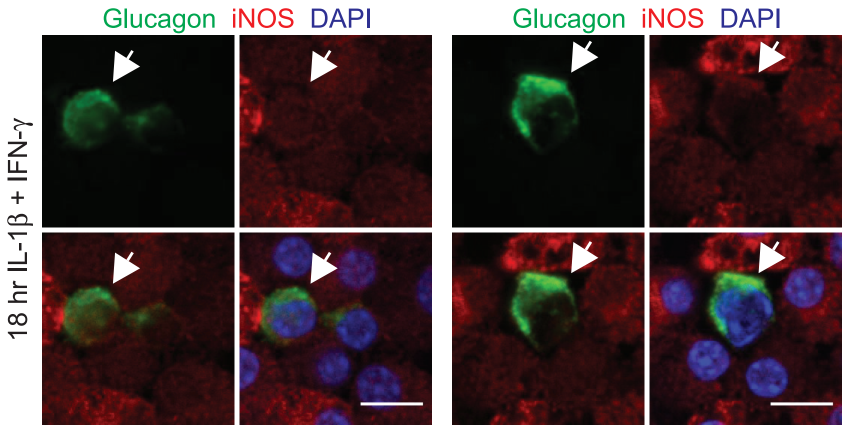

Figure S4: Colocalization of glucagon and iNOS in dissociated islets. Immunofluorescence images showing examples of dissociated mouse islet cells with co-localization of glucagon and iNOS protein following 18 hr cytokine treatment (10 U/mL IL-1β + 150 U/mL IFN-γ). Arrowheads indicate glucagon-containing cells also containing iNOS. Based on these images, iNOS expression in glucagon-containing cells in response to cytokine treatment is only slightly above background level. Scalebar = 10 μm.

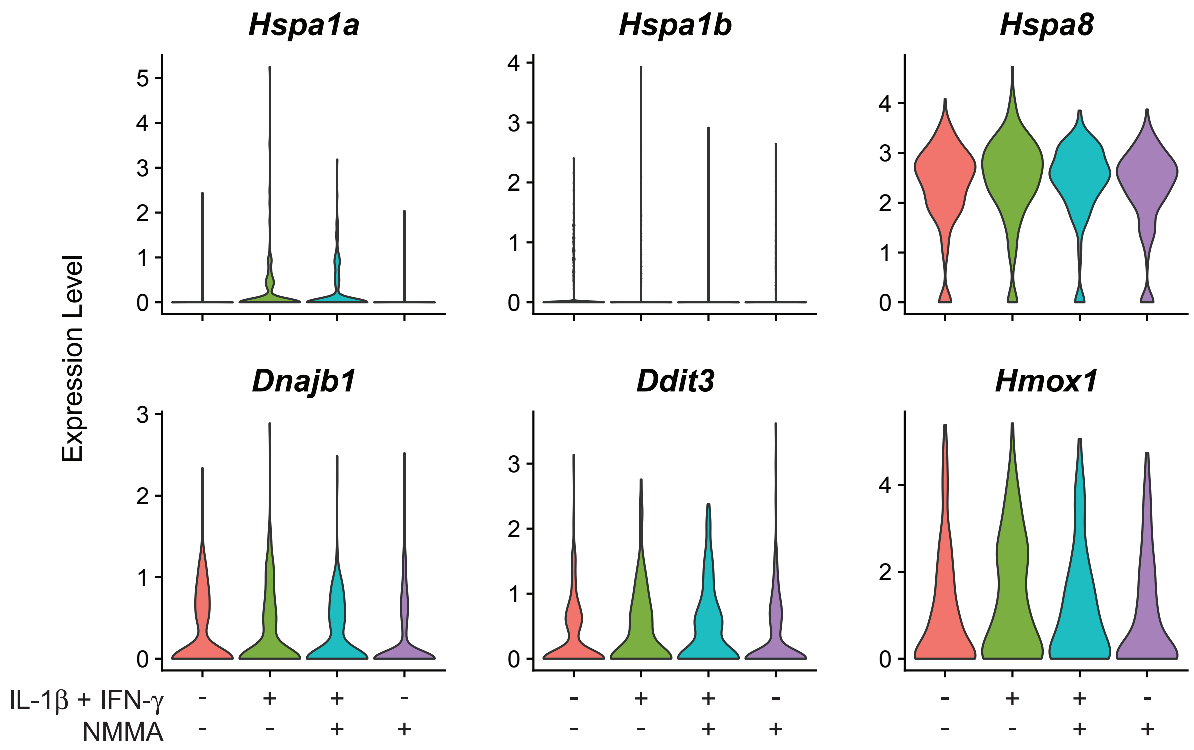

Figure S5: Expression of nitric oxide-dependent genes in islet non-endocrine cells. Violin plots showing the expression levels of selected genes that are regulated by nitric oxide in endocrine cells that do not demonstrate nitric oxide-dependent regulation in non-endocrine cells. Concentrations are as follows: 10 U/mL IL-1β, 150 U/mL IFN-γ, 2 mM NMMA.

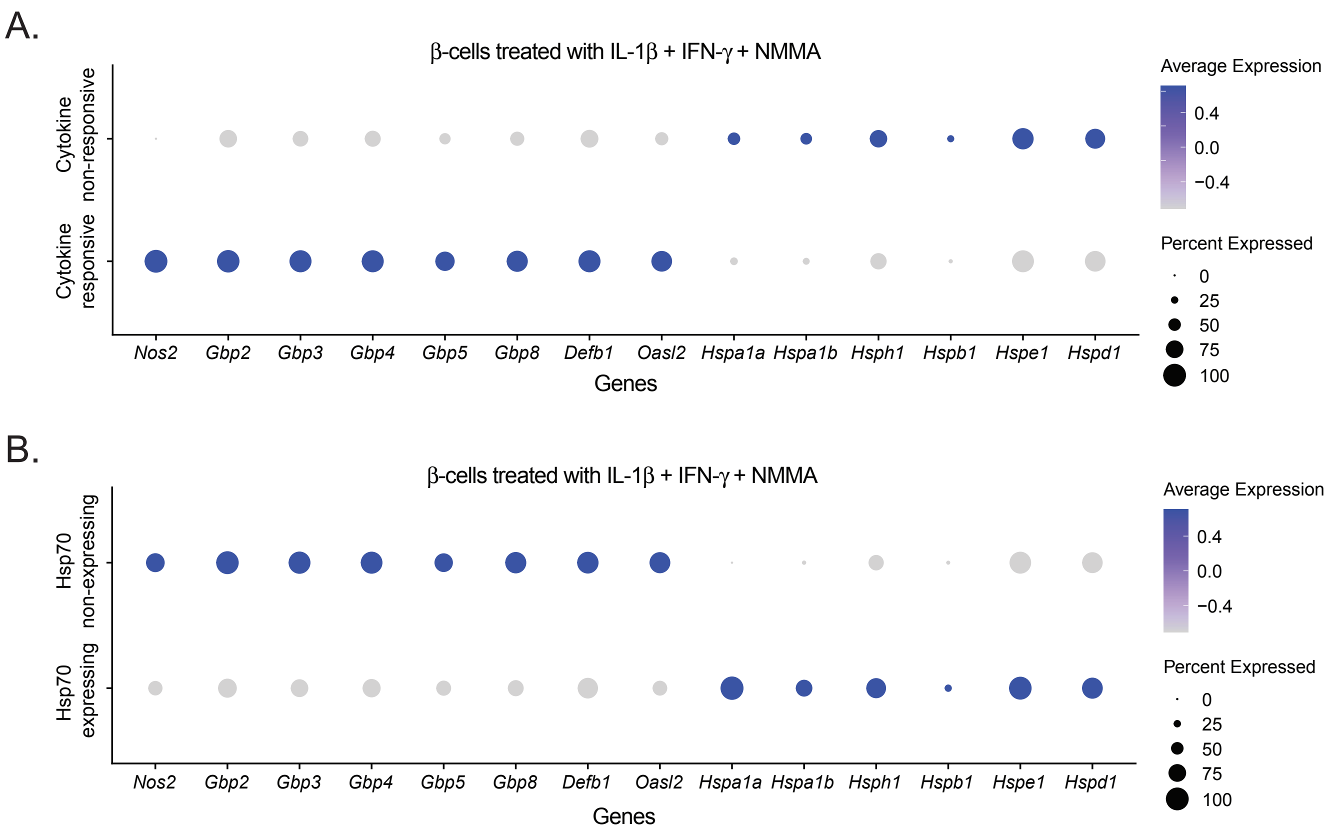

Figure S6: Cellular stress is negatively associated with cytokine signaling. (A) Dot plot depicting enrichment of selected genes between cytokine responsive (*Nos2*-expressing) and cytokine non-responsive (*Nos2* non-expressing) β-cells treated with IL-1β and IFN-γ + NMMA for 18 hr. **(B)** Dot plot depicting enrichment of selected genes between *Hspa1a*-expressing and *Hspa1a* non-expressing β-cells treated with IL-1β and IFN-γ + NMMA for 18 hr.

Stancill et al. Supplemental Tables

Table S1: Primer sequences

Table S2: Enriched genes in each cluster shown in Fig 1B.

Table S3: Differential expression analysis of β-cells in response to each treatment.

Table S4: Differential expression analysis of non-β endocrine cells in response to each treatment.

Table S5: Differential expression analysis of α-cells in response to each treatment.

Table S6: Differential expression analysis of δ-cells in response to each treatment.
